# The complete mitochondrial DNA sequence of Yimeng black goat (*Capra hircus*) and its potential application in mutton discrimination

**DOI:** 10.1080/23802359.2020.1773337

**Published:** 2020-06-02

**Authors:** Ke Liu, Ying Jin, Feng-Yan Zhang, Yan-Zhen Zhang, Xian-Qing Quan, Qing-Dian Han, Ling-Xiao Liu, Yun-Guo Liu, Shen-Jin Lv, Xiao-Ming Qu

**Affiliations:** a College of Life Sciences and Technology, Xinjiang University, Urumqi, China; b College of Life Sciences, Linyi University, Linyi, China; c Qingdao Customs District P.R. China, Qingdao, China; d Qingdao Institute for Food and Drug Control, Qingdao, China; e Linyi Academy of Agricultural Sciences, Linyi, China; f Liaoning Center for Disease Control and Prevention, Shenyang, China

**Keywords:** Yimeng black goat, *Capra hircus*, mitochondrial genome

## Abstract

Yimeng black goat is one of the national breeds of geographical indication in China and is one of the key protected local livestock and poultry breeds of Shandong province. The complete mitochondrial genome sequence of Yimeng black goat was investigated in this study (GenBank accession no. MT134111). The mitogenome (16,640 bp) consisted of a non-coding control region (D-loop region), two ribosomal RNA (rRNA) genes, 13 protein-coding genes (PCGs), and 22 transfer RNA (tRNA) genes. The complete mitochondrial genome sequence and the neighbour-joining tree of the Yimeng black goat would contribute to further study in genetic mechanism and phylogenomic research of goats.

Yimeng black goat (*Capra hircus*), an indigenous breed of Shangdong province in Eastern China, is mainly stocked in the higher altitudes of Mengshan, Lushan in Yimeng mountain (Yue et al. 2017). Good meat quality with low cholesterol content, high slaughter percentage and reproductive rate, and resistance against common diseases, special tolerance of adverse environmental conditions, and well adaptability to coarse feed are some of the outstanding features of Yimeng black goat. Due to the impact of breeding methods and the introduction of other breeds of goat and sheep, the number of Yimeng black goat has gradually decreased (Yang et al. 2014).

In this study, the complete mitochondrial genome of Yimeng black goat was sequenced and characterized in detail. The sample was collected from Mengyin City (35°43′N, 117°57′E), Shandong Province, China in January 2020. The specimen of Yimeng black goat, named as YimengBG-01, was stored in the College of Life Sciences, Linyi University, Linyi, China. Total genomic DNA was extracted from Yimeng black goat muscle according to Liu et al. (2012, 2014). The complete mitochondrial genome was sequenced using a shotgun approach and assembled with NOVOPlasty (Dierckxsens et al. 2016). The genome was annotated using MITOS web server (Bernt et al. 2013). The DNA sequence was analyzed using MEGA 7.0 (Kumar et al. 2016). Protein-coding genes were analyzed by ORF Finder (http://www.ncbi.nlm.nih.gov/gorf/gorf.html) using the vertebrate mitochondrial code. The tRNA genes were identified by ARWEN (Laslett and Canback 2008) and tRNA-scan SE (Chan and Lowe 2019).

The complete mitochondrial genome of Yimeng black goat (GenBank accession no. MT134111) is 16,440 bp in length, of which 15,359 nucleotides are coding DNA, and 1187 nucleotides are non-coding DNA. The total base composition of the mitochondrial genome is 33.52% A, 27.31% T, 13.13% G, and 26.04% C, and an A + T (60.83%)-rich feature occurs in the Yimeng black goat. To investigate the nucleotide bias, skew for a given strand was calculated as (A-T)/(A + T) or (G-C)/(G + C) (Perna and Kocher 1995). The AT and GC skews for the Yimeng Black goat mitochondrial genome were 0.102 and −0.330, respectively; this finding indicated that the strand that encoded genes contained more A and C than T and G, and this skew was evidence of codon usage bias.

The complete mitochondrial genome consists of 37 genes and a control region (D-loop). The 37 genes include 22 tRNA genes, 13 protein-coding genes, and two rRNA genes, which is consistent with the distribution and composition of the mitochondrial genome of other vertebrates (Parma et al. 2003; Sun et al. 2015; Chen et al. 2016; E et al. 2016; Jia and Wei 2016; Liu et al. 2016; Li, Liu, Sui, et al. 2019; Li, Liu, Zhang, et al. 2019). A neighbor-joining phylogenetic tree constructed from the complete mitochondrial genomes of 18 different species and breeds of *Caprinae* and one species of *Antilopinae* is shown in Figure 1. There are 12 overlapping regions (total 94 bp) and 15 intergenic spacers (total 69 bp) among the genes. The 13 protein-coding genes are NADH dehydrogenase subunits (*ND1*, *ND2*, *ND3*, *ND4*, *ND4L*, *ND5*, and *ND6)*, cytochrome c oxidase subunits I, II, and III (*COX1*, *COX2*, and *COX3*); ATP synthase subunits *ATPase*6 and *ATPase* 8 and cytochrome (*Cytb*) with the total length of 11,414 bp. Of these genes, except *ND2*, *ND3,* and *ND5* protein-coding genes having ATA as the start codon, *ND4L* using the start codon GTG, while the other nine protein-coding genes use the start codon ATG. When it comes to stop codons, six genes (*COX1*, *ATPase8*, *ATPase6*, *ND4L*, *ND5*, and *ND6*) terminate with TAA, whereas *Cytb* terminate with AGA. In addition, other six genes (*ND1*, *ND2*, *COX2*, *COX3*, *ND3*, and *ND4*) terminate with an incomplete stop codon ‘T––’ that is the 5′ terminal of the adjacent gene, which presumptively formed a complete stop codon by post-transcriptional polyadenylation (Anderson et al. 1981). All the mitogenome genes were encoded on the H strand except for *ND6* and eight tRNA genes (*tRNA^Gln^, tRNA^Ala^, tRNA^Asn^, tRNA^Cys^, tRNA^Tyr^, tRNA^Ser^, tRNA^Pro^ and tRNA^Glu^*). The *12S rRNA* (956 bp) gene and *16S rRNA* (1571 bp) gene were located between the *tRNA^Phe^* and *tRNA^Leu^* genes and separated by the *tRNA^Val^* gene. The 22 tRNA genes ranged in length from 60 (*tRNA^Ser^*) to 75 bp (*tRNA^Leu^*) and were the same with other goats (Ran et al. 2016; Tang et al. 2016; Zhang et al. 2016). The D-loop region locates between *tRNA^Pro^* and *tRNA^Phe^* with a length of 1012 bp. Moreover, the small non-coding region, a putative origin of the L strand replication, was located between *tRNA^Asn^* and *tRNA^Cys^* genes in the length of 32 bp. The data would promote further investigations of phylogenetic relationships within *C. hircus* and show a potential application in mutton discrimination.

**Figure 1.**
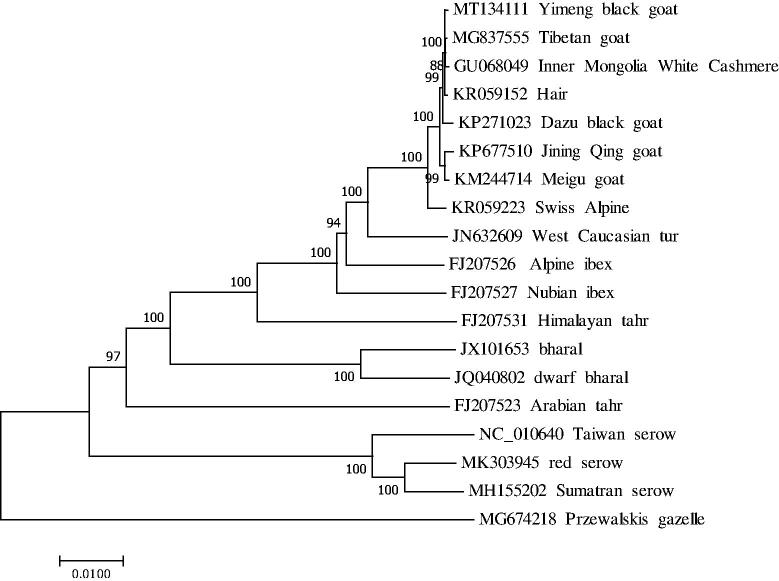
Neighbor-joining tree based on combining the complete mitochondrial genome sequences of 19 species by using MEGA 7.0. Bootstrap values based on 1000 replicates are shown at branch nodes.

## Data Availability

The data that support the findings of this study are openly available in GenBank at https://www.ncbi.nlm.nih.gov, reference number MT134111.
